# Chemically Surface Tunable Solubility Parameter for Controllable Drug Delivery—An Example and Perspective from Hollow PAA-Coated Magnetite Nanoparticles with R6G Model Drug

**DOI:** 10.3390/ma11020247

**Published:** 2018-02-06

**Authors:** Quanguo He, Jun Liu, Jing Liang, Xiaopeng Liu, Du Tuo, Wen Li

**Affiliations:** School of Life Science and Chemistry, Hunan University of Technology, Zhuzhou 412007, China; hequanguo@126.com (Q.H.); junliu@hut.edu.cn (Ju.L.); liangjingabbey@126.com (Ji.L.); amituo321@163.com (X.L.); dutuo99@hut.edu.cn (D.T.)

**Keywords:** hollow Fe_3_O_4_ NPs, Fe_3_O_4_/PAA composite NPs, solubility parameter, controlled release, drug delivery systems

## Abstract

Solubility parameter-dependent drug releasing property is essential in practical drug delivery systems (DDS), and how to combine magnetic nanoparticles(NPs) and suitable polymer coating towards DDS is always a crucial and valuable challenge in biomedical application. Herein, a controllable drug delivery model with a surface having a chemically tunable solubility parameter is presented using hollow magnetite/polyacrylic acid (Fe_3_O_4_/PAA) nanocomposites as nanocarrier towards DDS. This composite is prepared by simply coating the modified hollow Fe_3_O_4_ with PAA. The coating amount of PAA onto the surface of Fe_3_O_4_ (measured by TGA) is about 40% (*w*/*w*). Then, Rhodamine 6G (R6G) is selected as model drug in drug delivery experiment. The efficiency of drug loading and drug release of these Fe_3_O_4_/PAA nanocarriers are evaluated under various temperature, solvent and pH values. As a result, the best drug releasing rate was achieved as 93.0% in pH = 7.4 PBS solution after 14 h. The releasing efficiency is 86.5% in acidic condition, while a lower releasing rate (30.0%) is obtained in aqueous solution, as different forms (polyacrylic acid and polyacrylate) of PAA present different solubility parameters, causing different salt and acid effects in various solvents, swelling property of PAA, and binding force between PAA and R6G. Therefore, by changing the solubility parameter of coating polymers, the drug delivery properties could be effectively tuned. These findings prove that the DDS based on magnetic particle cores and polymer encapsulation could efficiently regulate the drug delivery properties by tuning surface solubility parameter in potential cancer targeting and therapy.

## 1. Introduction

In recent decades, drug delivery system (DDS) based on nanomaterials has become an important potential method for cancer therapy in biomedical field. By combining the targeting and controlled drug release properties of these composite nanocarriers, the cancer therapeutic effect is improved because of the efficient utilization of these drugs with high concentration in cancer-related locus. The target property of nanocarriers is the prerequisite for delivering anti-cancer drug to desirable site in vivo. It could be realized by various methods, such as magnetic-guided targeting and specific ligands binding target (including antibody, polypeptide, sugar chain, and nucleic acid adapter). As one of the best magnetic materials, Magnetite (Fe_3_O_4_) NPs are widely used in target DDS [1,2,3]. The magnetic property endows the nanocarriers with more specific targeting of cancer disease site under external magnetic field application. Interestingly, the Fe_3_O_4_ NPs with internal mesoporous/hollow structures or with holes and cavities facilitate more drug loading for DDS. DDS applications of magnetic NPs requires not only the inherent magnetic performances improvement [4,5,6,7], but also their surface modification to enable multifunctional responses to various external changes [8,9,10,11]. Sensitive polymer modification is an effective alternative to overcome the above-mentioned problem; it enables the nanocarriers to act as multifunctional drug delivery carriers under different tumor circumstances [12,13]. These polymer-coated Fe_3_O_4_ NPs could improve the biocompatibility as well as control the drug release rate effectively. Moreover, different functional polymers could provide different controlled release mechanisms, such as pH, temperature, chemical addition, or light triggered drug delivery systems [14]. For example, Zhao and co-workers synthesized a kind of yolk–shell magnetic composite NPs, which contained polymethacrylic acid (PMAA) coating and Fe_3_O_4_ NPs core. The composite NPs acted as a magnetic-pH response system. The in vitro model drug delivery of Fe_3_O_4_/PMAA NPs is also investigated with ceftriaxone sodium. The cavity of the yolk–shell structure could greatly improve the drug loading ability, and the polymer layer of PMAA could reduce the releasing rate under the acid condition [15].

However, for all these controlled releasing mechanisms, solubility parameter regulation is also important method according to the “like dissolves like” theory, and it is often ignored in DDS. Few investigations are focused on DDS using the principle of solubility parameter regulation of polymer coating. In our opinion, to attain controllable drug release, tunable solubility parameter surface in nanocarrier of DDS could be an ideal solution to address such a challenge. Generally, the drug releasing rate is directly determined by dissolvability of polymers and drugs in solvent. Good dissolvability in a solvent could improve the drug release rate. A similar solubility parameter of polymer, solvent or drugs promote the dissolvability of polymer and drugs in solvent [16,17]. Therefore, in the drug loading process, drugs could be dispersed uniformly in polymer as well as in the hollow inside structure of nanocarriers. Moreover, during drug release process, these polymers swell well in solvent, and drugs are easily released into solvent. Consequently, the drug release property could be regulated by the solubility parameter of coated polymers. However, few studies are focused on this influence of solubility parameter on drug delivery property. Polyacrylic acid (PAA) is a acidic polymer, which is usually used in biomedical field [18]. Moreover, PAA is a pH responsive polymer, and the pH values could affect the drug loading and releasing properties greatly. The main reason arises from different forms of PAA in solvent being able to induce different dissolvability of drug, and the stretch performance of PAA is a main factor in drug delivery process.

Herein, a drug delivery system example based on PAA coated hollow Fe_3_O_4_ NPs is presented. The influence of solubility parameter changes of PAA on the alteration of drug loading and releasing properties is investigated, calculated and compared. The hollow structure of Fe_3_O_4_ is aimed for drug loading [4,5,6,7], and the PAA coating is presented as a controlled drug release material with chemically tunable solubility parameter. R6G is selected as model drug for loading and releasing investigations of these hollow Fe_3_O_4_/PAA NPs. The influence factors for these drug loading and releasing performance are also investigated. Finally, the drug release mechanism based on the solubility parameter alteration of these hollow Fe_3_O_4_/PAA NPs are discussed and compared carefully. In contrast with conventional pharmaceutical design and DDS design availability, the solubility parameter sets a fine example and offers a unique perspective for surface tunable polymer coating towards controllable DDS which is versatile for both hydrophilic and hydrophobic drugs with similar solubility parameter. Meanwhile, as a rough prototype, it will encourage and inspire more research efforts for practical solubility parameter-driven DDS.

## 2. Material and Methods

Ferric chloride hexahydrate (FeCl_3_·6H_2_O), anhydrous sodium acetate (NaAc), ethylene glycol (EG), ethanol (99.7%), sodium phosphate (Na_2_HPO_4_), sodium dihydrogen phosphate (NaH_2_PO_4_), sodium hydroxide (NaOH), hydrogen chloride (HCl) and other chemical reagents with analytical grade were purchased from Sinopharm Chemical Reagent Co. Ltd. (Shanghai, China) 3-ammonia propyltriethysilane (APTES), polyacrylic acid (PAA, WM 3000), 1-(3-Dimethylamino propyl)-3-ethyl carbodiimide hydrochloride (EDC·HCl), *N*-Hydroxy succinimide (NHS), and Rhodamine 6G (R6G) were purchased from Shanghai Aladdin Reagents Co. Ltd. (Shanghai, China). Millipore water (18.2 MΩ cm at 25 °C) was used throughout all experiments.

Field emission Transmission electron microscopy (TEM) images were taken using a Hitachi JEM-1230 transmission electron microscope (JEOL, Tokyo, Japan) at an accelerating voltage of 200 kV. Scanning electron microscopy (SEM) images were obtained using a Hitachi S-3000 (Hitachi, Tokyo, Japan). X-ray powder diffraction (XRD) measurements were performed on a Bruker Advanced-D8 (Bruker AXS GmbH, Karlsruhe, Germany). Nanoparticle size analyzer patterns were taken at Malvern Zetasizer Nano S90 (Malvern, Worcestershire, UK). The HH-15 vibrating sample magnetometer (VSM) (Nanjing University Instrument Factory, Nanjing Institute of Electronics Ding one hundred) was used in the magnetic measurement. The FT-IR spectra were measured by FT-IR Spectrometer (Vector-22, Bruker Analytic GmbH, Hamburg, Germany). Thermogravimetric analyzer (TGA) (Q50, TA, New Castle, DE, USA) was used to measure the amount of coated PAA shell, Ultraviolet–visible spectrophotometer (UV-Vis) (T-1901, Purkinje General Corporation, Beijing, China).

### 2.1. Synthesis of Hollow Fe_3_O_4_ NPs

First, 5 mmol of FeCl_3_·6H_2_O were dissolved in 40 mL of EG to form a clear brown solution, and then 40 mmol of anhydrous NaAc were added. The mixture was vigorously mixed by ultrasonication (10 min) for formation of a homogeneous solution. Then, the solution was transferred into a Teflon-lined stainless steel autoclave (100 mL capacity) for hydrothermal treatment at 200 °C for 20 h. After the autoclave was allowed to cool down to room temperature, the precipitate was collected by magnetic separation and washed by water and ethanol several times under sonication. Finally, the products were dried under vacuum at room temperature.

### 2.2. The Surface Coating and Decoration of Hollow Magnetic Composite Particles

Forty milligrams of Fe_3_O_4_ NPs were suspended into 100 mL of ethanol solution, and then 0.5 mL of APTES solution ethanol (5% *v*/*v*) were added into the into above solution dropwisely. After several minutes, 1 mL of pure water was then added dropwisely. The mixture was stirred for 3 h. The APTES-modified Fe_3_O_4_ NPs were collected by magnet, and washed by ethanol and deionized water three times, respectively. Then, 100 μL of PAA solution was first added to another 5 mL of buffer solutions (pH = 6.0) with 0.5 mmol EDC·HCl solutions. After this solution was sonicated for 10 min, 0.5 mmol of NHS solutions were added under ultrasound for another 30 min. Ten milligrams of above APTES-modified Fe_3_O_4_ NPs were finally added under stirring for 4 h. Finally, the precipitate was collected by magnetic separation and washed several times under sonication with water and ethanol and dried under vacuum at room temperature before characterization and application.

### 2.3. The Preparation of Standard Curve of Rhodamine 6G

Rhodamine 6G (R6G) was used as simulative standard loading drug in this test. To obtain the relationship between concentration and absorbance, the standard curve determination of R6G was carried out. Specifically, 0.0072 g of R6G were dissolved into 1 L of pure water to form 0.15 × 10^−4^ mol/L of homogenous solution. Then, the prepared R6G solution was diluted to 5 precise concentration (0.03, 0.06, 0.09, 0.12, and 0.15 × 10^−4^ mol/L) to acquire concentration gradients. The absorbances of above R6G solutions were determined by UV-Vis spectrometer at wavelength range of 200–700 nm. Consequently, the standard curve equation of the relationship between concentration and absorption was obtained.

### 2.4. Drug Loadings and Releasing Tests of Fe_3_O_4_/PAA

For the drug loading test of Fe_3_O_4_/PAA composite NPs, in typical process, 0.01 g of Fe_3_O_4_/PAA NPs were dispersed in 10 mL of phosphate buffered solution (PBS) by ultrasonication for 30 min. Then, 10 mL of R6G solution (1 × 10^−3^ mol/L) were added into the above suspension. In all cases, Fe_3_O_4_/PAA composite NPs reached the adsorption equilibrium within 30 min at 30 °C. After the magnetic separation of drug loaded Fe_3_O_4_/PAA composite NPs, the supernatants were determined by UV-Vis spectrometer at 526 nm in the range of 200–700 nm. Moreover, the absorption time, temperature, pH and concentration of R6G are investigated to acquire the best drug loading amount. 

The in vitro drug releasing test were conducted by using drug loaded Fe_3_O_4_/PAA composite NPs under different pH environments. Typically, 0.01 g drug loaded Fe_3_O_4_/PAA composite NPs were dispersed into 100 mL of PBS. This solution was maintained at 37 °C under slight shaking (50 rpm/min). Then, the absorbance of supernatant was determined every 20 min. As the release time increased, the sample intervals were prolonged to 40 min, 1 h and 2 h gradually. Thus, the drug releasing rates of these drug loaded Fe_3_O_4_/PAA composite NPs were calculated accordingly.

## 3. Result and Discussion

### 3.1. The Principle of the Preparation of Fe_3_O_4_/PAA Composite Magnetic NPs

**Figure 1:**
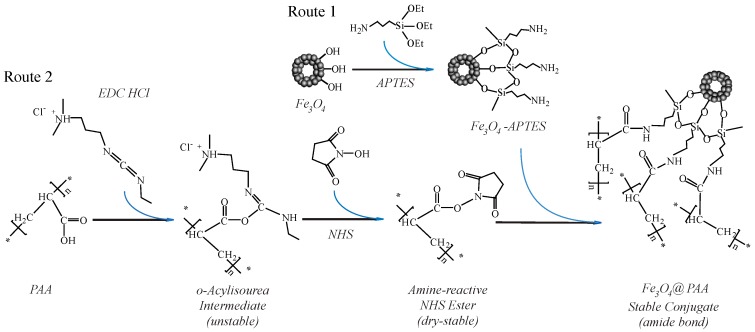
Schematic illustration of the process of Fe_3_O_4_/PAA composite magnetite hollow spheres formation. Abbreviations: Fe_3_O_4_/PAA, PAA coated Fe_3_O_4_.

The hollow Fe_3_O_4_ NPs are coated with PAA to form the composite nanostructures by surface modification and chemical crosslinking reaction as depicted in Figure 1. Firstly, the surface of hollow Fe_3_O_4_ NPs are modified with amino group (–NH_2_) terminal by using APTES as coupling agent. After hydrothermal reaction of magnetite formation, there are many hydroxyl (–OH) groups present on the surface of Fe_3_O_4_ NPs. The hydrolytic reaction could initiate with these hydroxyl groups after APTES and water addition. The chemistry principle is shown in Figure 1 (Route 1). Then, the carboxyl (–COOH) groups could be activated by EDC·HCl and NHS. The active *O*-acylisourea intermediate is formed by firstly adding of EDC·HCl. However, the *O*-acylisourea intermediate is unstable in aqueous solutions; its failure to react with an amine results in hydrolysis of the *O*-acylisourea intermediate. The carboxyl is regenerated as a result, and an *N*-unsubstituted urea is released. Thus, the subsequently added NHS could replace the EDC·HCl rapidly, and the stable NHS-ester is formed. The specific route is shown in Figure 1 (Route 2). After adding the amino-modified Fe_3_O_4_ NPs, stable NHS-ester is easily displaced by nucleophilic attack from primary amino groups (from amino-modified Fe_3_O_4_ NPs) in the reaction mixture. Finally, the stable amide bonds are formed by combination of the primary amines and the original carboxyl groups (as shown in Figure 1 (Route 2)). Therefore, the PAAs are coated on the surface of hollow Fe_3_O_4_ NPs successfully.

### 3.2. The Morphologies of the Fe_3_O_4_/PAA Composited NPs

**Figure 2:**
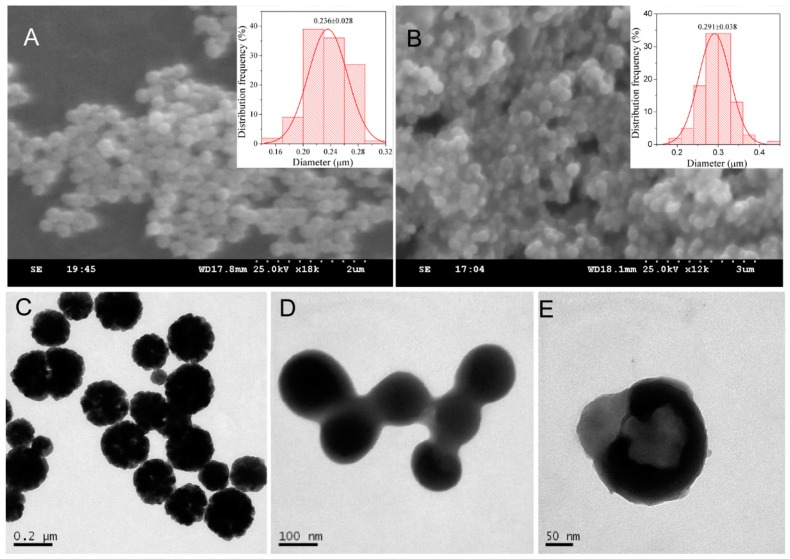
SEM and TEM images of: Fe_3_O_4_ before coating (**A**,**C**); and Fe_3_O_4_/PAA, after coating (**B**,**D**,**E**). Insets of (**A**,**B**) are their corresponding size distribution.

The morphologies of as-prepared hollow Fe_3_O_4_ NPs and Fe_3_O_4_/PAA composite NPs are characterized by SEM and TEM techniques. Figure 2A,B shows SEM images of hollow Fe_3_O_4_ NPs and Fe_3_O_4_/PAA composite NPs, respectively. As shown in Figure 2A, the good dispersity of the hollow Fe_3_O_4_ NPs is presented. After coating with PAA shell, the dispersity of these Fe_3_O_4_/PAA composite NPs becomes worse because the coated PAAs easily aggregate with each other. The insets are the size distribution of hollow Fe_3_O_4_ NPs and Fe_3_O_4_/PAA NPs, and the average sizes are 236 and 291 nm, respectively. After PAA coating, the average sizes of Fe_3_O_4_/PAA NPs are enhanced, and the size distribution range is wider than for pure parent Fe_3_O_4_ NPs. Figure 2C is a TEM image of hollow Fe_3_O_4_ NPs. Evidently, these Fe_3_O_4_ NPs show the cavity structures under hydrothermal ripening process. After coating with PAA shell, the morphology of Fe_3_O_4_/PAA composite NPs is different from the parent Fe_3_O_4_ NPs. The PAA adhesive layers are obviously characterized by smooth surface, and the hollow structure of internal Fe_3_O_4_ NPs becomes obscure (Figure 2D). Furthermore, the amplified TEM image of single Fe_3_O_4_/PAA nanoparticle is presented in Figure 2E. The core–shell structure is more evident, and the hollow structure of Fe_3_O_4_ NPs could also be observed. These results indicate that the Fe_3_O_4_/PAA composite NPs are synthesized successfully. 

### 3.3. The Structure and Element Analysis of the Fe_3_O_4_/PAA Composited NPs

**Figure 3:**
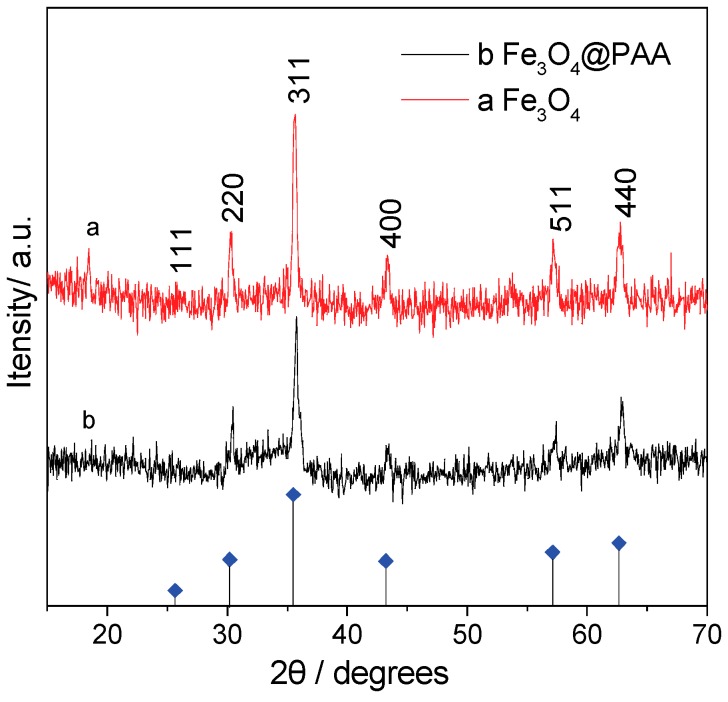
XRD patterns of: Fe_3_O_4_ (**a**); and Fe_3_O_4_/PAA composite NPs (**b**).

The crystal structures of these hollow Fe_3_O_4_ NPs and Fe_3_O_4_/PAA composite NPs are also investigated by XRD. In Figure 3, curve (a) and curve (b) are the XRD patterns of hollow Fe_3_O_4_ NPs and Fe_3_O_4_/PAA composite NPs, respectively. Moreover, the peak positions of XRD patterns in both two curves are almost the same, and the characteristic peaks located at 30.4°, 35.7°, 43.2°, 53.7°, 57.3° and 62.8° could be indexed to (220), (311), (400), (400), (511) and (440) facets of inverse spinel magnetite (standard PDF Card NO. JSPDS 01-1111, *α* = 8.393 Å), respectively. However, the intensity of these peaks for Fe_3_O_4_/PAA composite NPs are lower than those of parent hollow Fe_3_O_4_ NPs, which indicates that the PAA coating could reduce the XRD intensity of hollow Fe_3_O_4_ NPs effectively.

**Figure 4:**
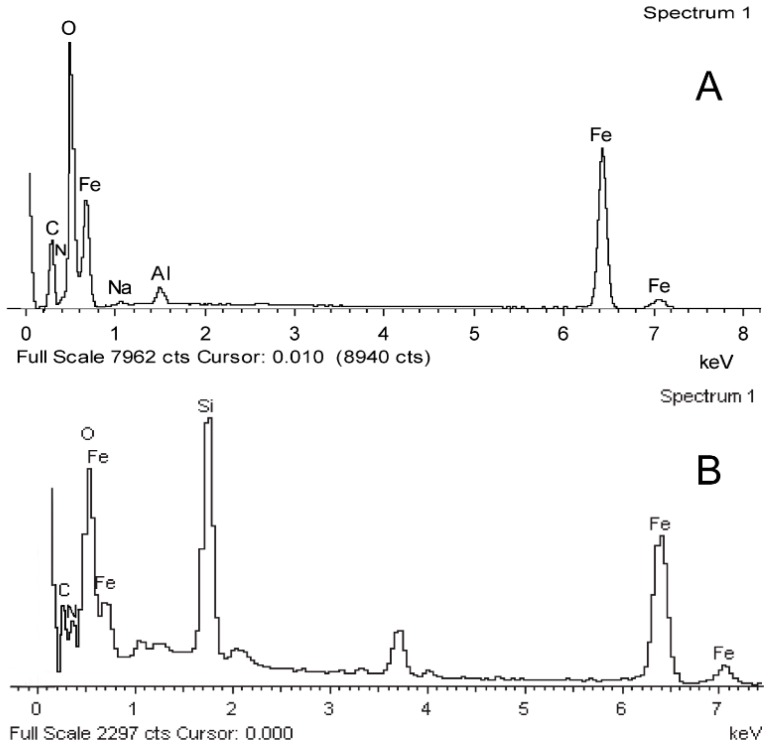

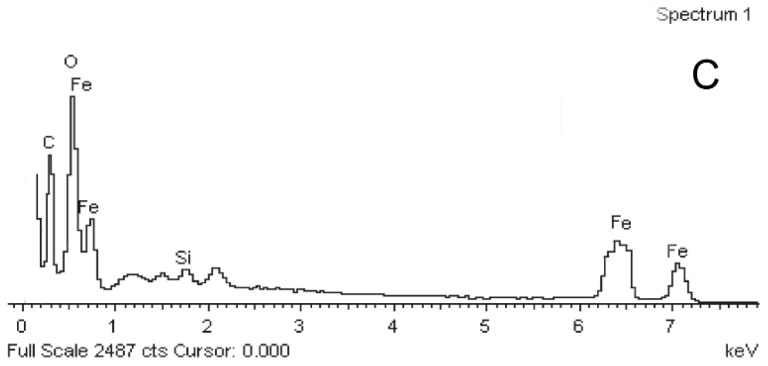
EDX patterns of: Fe_3_O_4_ (**A**); APTES-Fe_3_O_4_ (**B**); and Fe_3_O_4_/PAA composite NPs (**C**). Abbreviations: Fe_3_O_4_/PAA, PAA coated Fe_3_O_4_; APTES-Fe_3_O_4_, (3-Aminopropyl)-triet-hoxysilane modified Fe_3_O_4_; EDX, energy dispersive X-ray spectroscopy.

Moreover, the element analysis of these samples is also investigated by EDX technique. Figure 4A–C shows the EDX spectra of hollow Fe_3_O_4_ NPs, APTES modified Fe_3_O_4_ NPs and Fe_3_O_4_/PAA composite NPs, respectively. Obviously, in Figure 4A, Fe and O could be found in the EDX, and Na, C and N come from reactants, while Al is from the aluminum substrate. As shown in Figure 4B, after modification with APTES, Si emerges because of silane coupling agent of APTES. Moreover, this sharp peak of Si is decreased after PAA coating, as shown in Figure 4C. Meanwhile, C is enhanced in Fe_3_O_4_/PAA composite NPs sample, which is probably due to the PAA polymer’s coating, as it shields the inner core elements. All the above results indicate that the hollow Fe_3_O_4_ NPs are coated by PAA shell to form a composite system as desired.

### 3.4. The Magnetic Properties of the Fe_3_O_4_/PAA Composited NPs

**Figure 5:**
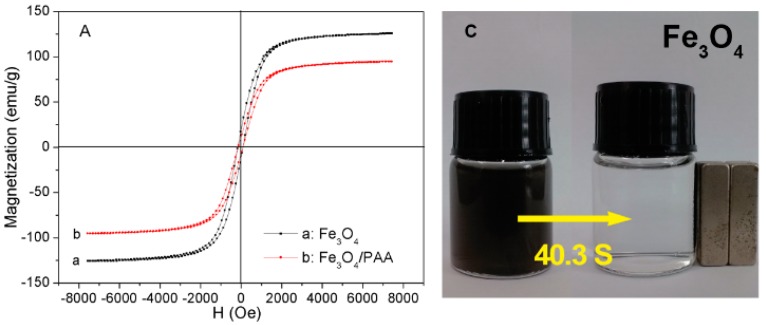

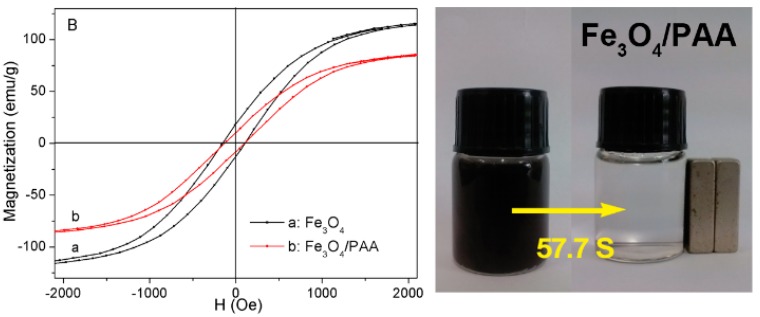
The hysteresis loops at T = 300 K of: Fe_3_O_4_ (b) and Fe_3_O_4_/PAA (a) (**A**); magnified part of the curves in (**A**,**B**); and photograph showing magnetic isolation (**C**).

The magnetization curves of hollow Fe_3_O_4_ NPs and Fe_3_O_4_/PAA composite NPs are shown in Figure 5A. The saturation magnetization values of hollow Fe_3_O_4_ NPs and Fe_3_O_4_/PAA composite NPs are 125.0 (curve a) and 94.6 (curve b) emu/g, respectively. The present coercivity (*Hc*) values of both hollow Fe_3_O_4_ NPs and Fe_3_O_4_/PAA composite NPs in Figure 5B are less than 100 Oe (A/m), indicating their very good soft magnetism. In addition, as compared with that of the hollow Fe_3_O_4_ NPs, the saturation of that PAA coating is decreased because the nonmagnetic PAA could reduce the weight of Fe_3_O_4_ NPs per unit volume [19,20]. This result indicates that the PAA are successfully coated on the surface of magnetic NPs. Figure 5C shows photographs of the suspension of Fe_3_O_4_ NPs and Fe_3_O_4_/PAA composite NPs with and without of an external magnet contact. The nanospheres can easily disperse in ethanol solution to form a black suspension, and are drawn from the solution to the sidewall of the vial by an external magnetic field application within 1 min. The magnetic particles can be brought again back into the original solution by removing the external magnetic field application and then slightly agitating. However, the adhesion time of Fe_3_O_4_/PAA composite NPs is prolonged compared to Fe_3_O_4_ NPs. It implies that the PAA coating weakens the magnetism of the parent cores of magnetite. This result is consistent with the magnetic characterization outcome. 

### 3.5. The FT-IR Spectra of the Fe_3_O_4_/PAA Composited NPs

**Figure 6:**
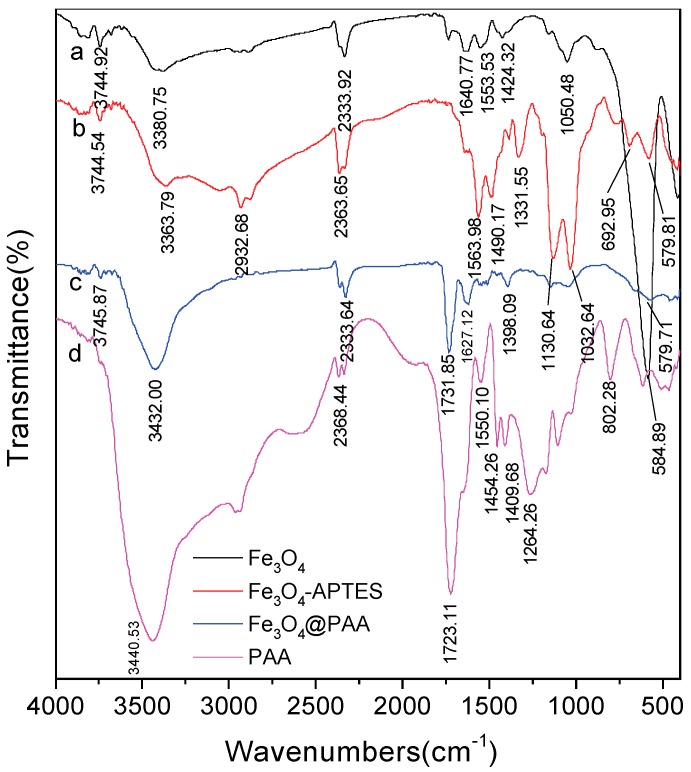
The FT-IR spectra of: bare Fe_3_O_4_ NPs (**a**); APTES- Fe_3_O_4_ (**b**); Fe_3_O_4_/PAA composite NPs (**c**); and PAA (**d**).

The PAA coated hollow Fe_3_O_4_ NPs are further confirmed by Fourier transform infrared (FT-IR) spectra. The FT-IR spectra of hollow Fe_3_O_4_ NPs, APTES modified Fe_3_O_4_NPs, Fe_3_O_4_/PAA composite NPs and pure PAA are presented in Figure 6a–d. As shown in Figure 6a, the characteristic peak of Fe_3_O_4_ NPs appears at 584.89 cm^−1^. In Figure 6b, after modification with APTES, the peaks at 1130, 1331.55, and 1563.98 cm^−1^ are assigned to stretching vibration of Si-O, C-N and N-H, respectively. As compared with FT-IR spectra of pure Fe_3_O_4_ NPs, this result proves that APTES is modified on the surface of Fe_3_O_4_ NPs. Moreover, a very strong band emerges at around 1627.12 cm^−1^ in curve c, which belongs to the stretching vibration of carbonyl (–C=O) bond in protonated carboxylate groups. This position is consistent with curve d (FT-IR spectra of pure PAA), indicating that the PAA exists in the Fe_3_O_4_/PAA composite NPs.

### 3.6. The Thermogravimetry of the Fe_3_O_4_/PAA Composited NPs

**Figure 7:**
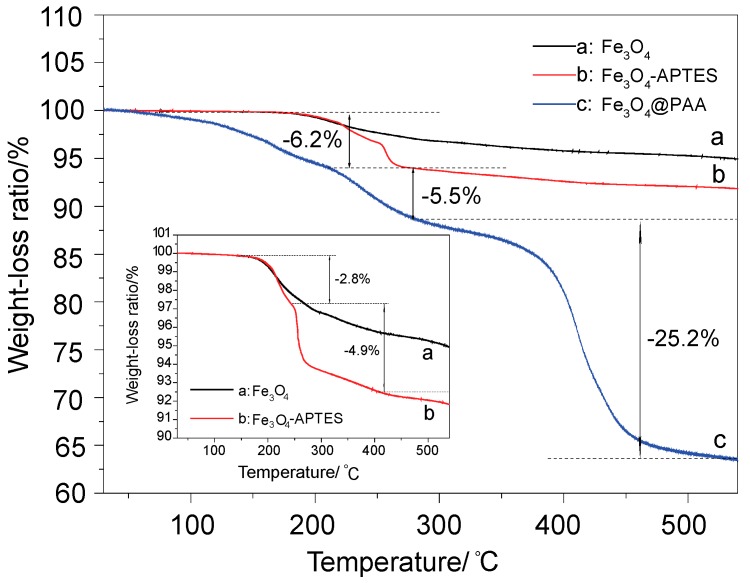
The thermogravimetric profile of: bare Fe_3_O_4_ NPs (**a**); APTES- Fe_3_O_4_ (**b**); and Fe_3_O_4_/PAA composite NPs (**c**).

The TGA analysis of these as-prepared samples are presented for understanding the amount of the organic coatings. Figure 7a–c shows the TGA analysis of hollow Fe_3_O_4_ NPs, APTES modified Fe_3_O_4_ NPs and Fe_3_O_4_/PAA composite NPs, respectively. Figure 7a shows the TGA curve of hollow Fe_3_O_4_ NPs, where about 5% of weight is lost after the temperature rises to 500 °C. The lost could be attributed to the burning of organic groups on the surface of Fe_3_O_4_ NPs and a little water evaporation. Figure 7b shows the TGA curve of APTES modified hollow Fe_3_O_4_ NPs, where about 8% of weight is lost. The weight losses of these two steps are 2.8% and 4.9%, respectively (the inset of Figure 7). A new weight losing step could be observed, which is ascribed to the loss of modified APTES shell. Figure 7c shows the TG curve of Fe_3_O_4_/PAA composite NPs, where three weight loss steps are included. About 5.6% of weight is lost in Step 1, which begins at 50–60 °C and might be due to PAA being water adsorbing material, i.e., a lot of water is in the Fe_3_O_4_/PAA composite NPs. About 5.5% of weight is lost in Step 2, which is almost the same as that in Figure 7b. Moreover, 25.2% of weight is lost in Step 3, indicating that the organic materials (PAA) are lost with the raising of temperature. The ratio of PAA shell to hollow Fe_3_O_4_ NPs in this Fe_3_O_4_/PAA composite NPs could be calculated as follows: 25.2%/(100% − 25.2% − 6.2% − 5.5%). About 40% (*w*/*w*) of PAA are coated onto the surface of hollow Fe_3_O_4_ NPs.

### 3.7. Drug Loadings Tests of the Fe_3_O_4_/PAA Composited NPs

**Figure 8:**
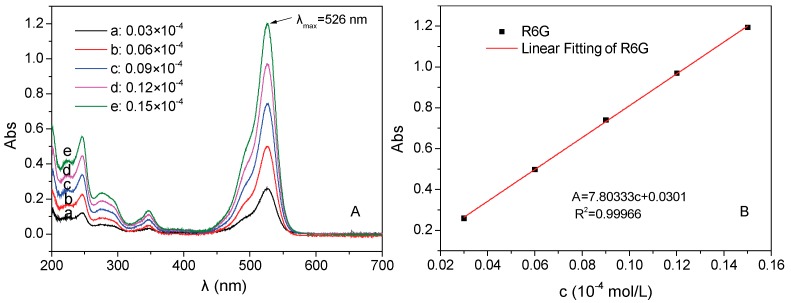
(**A**) UV-Vis spectra of the five groups of R6G with standard concentration (λ_max_ = 526 nm); and (**B**): the linear fitting of R6G.

**Figure 9:**
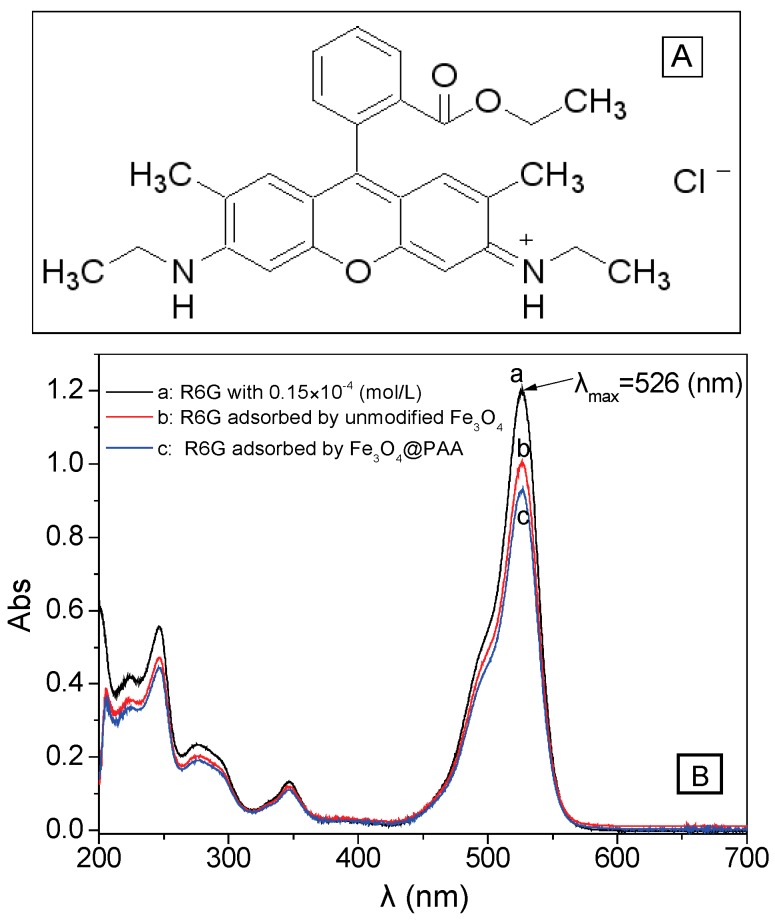
(**A**) Structure of R6G and (**B**) UV-Vis spectra of the three groups of R6G (λ_max_ = 526 nm): (**a**) absorbance of 0.15 × 10^−4^ mol/L of R6G; (**b**) absorbance of R6G adsorbed by hollow Fe_3_O_4_; and (**c**) absorbance of R6G adsorbed by Fe_3_O_4_/PAA NPs.

Firstly, the standard linear equation of R6G is measured to obtain the relationship between concentration and absorbance (Figure 8A). R6G solution is used as the simulative drug for the drug loading and releasing test process, because R6G possess an amino hydrochloride salt structure (Figure 9A), and is highly similar to the molecular weight and surface groups of many anticancer drugs. After measuring the UV-visible spectra of R6G, the linear fitting is presented in Figure 8B, and the standard linear equation is calculated as follows: A = 7.80333c + 0.0301 (R^2^ = 0.99966, where A is the absorbance and c is the concentration (unit: ×10^−4^ mol/L)). Moreover, pure hollow Fe_3_O_4_ NPs and Fe_3_O_4_/PAA composited NPs are used to adsorb and accommodate the R6G (c = 0.15 × 10^−4^ mol/L) at room temperature. As shown in Figure 9B, the absorbance intensities decrease after adding pure hollow Fe_3_O_4_ NPs (curve b) and Fe_3_O_4_/PAA composite NPs (curve c). After calculation, 1.00 g of unmodified hollow Fe_3_O_4_ NPs could adsorb 229.9 mg of R6G, while 1.00 g of Fe_3_O_4_/PAA composited NPs could adsorb 325.7 mg R6G, greatly enhancing the model drug loading. These phenomena indicate that the hollow Fe_3_O_4_ NPs show the capacity of drug loading, and, after coating of PAA shell, the amount of drug loading is increased correspondingly.

### 3.8. The Influence Factors for Adsorption Properties of the Fe_3_O_4_/PAA Composited NPs

**Figure 10:**
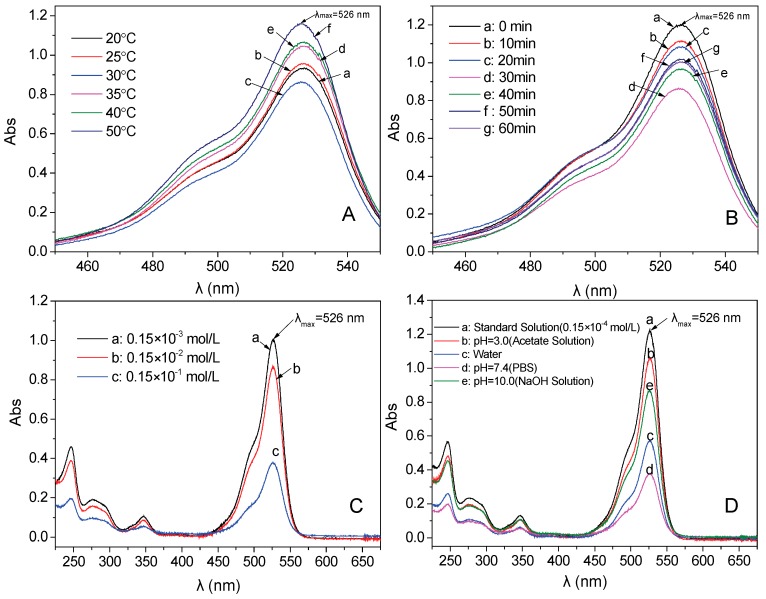
UV-Vis spectra of the supernatant after R6G adsorbing with Fe_3_O_4_/PAA for different conditions (λ_max_ = 526 nm): (**A**) temperature; (**B**) adsorptive time; (**C**) adsorptive concentration; and (**D**) pH.

The drug loading conditions (temperature, time, pH and concentration of drugs) are investigated to obtain the best loading condition. Firstly, six different temperatures are compared to understand the influence of temperature on the amount of drug loading (other condition: the concentration of R6G is 0.15 × 10^−2^ mol/L, mass of Fe_3_O_4_/PAA composited NPs is 0.010 g, pH is 7.4, and time is 30 min). After the loading process, the adsorption performances of supernatants are presented in Figure 10A. The intensity order of these absorbances obtained at 526 nm is: 50 °C > 40 °C > 35 °C > 25 °C > 20 °C > 30 °C. This result indicates that the largest absorbance is obtained under the temperature of 30 °C because the intermolecular motion became fierce with the higher temperature. The best drug loading amount is 408.5 mg of R6G per 1.00 g of Fe_3_O_4_/PAA composited NPs, and the loading results are listed in Table 1.

Then, six different adsorption times are compared to investigate the influence of absorption time on the amount of drug loading. The best adsorption temperature (30 °C) is used in this experiment. Other conditions are kept the same to the process mentioned above. The intensity order of these absorbances at 526 nm is: 0 min > 10 min > 20 min >50 min > 60 min > 40 min >30 min (Figure 10B). It indicates that 30 min is the best adsorption time because the desorption effect also occurs when the adsorption time is prolonged. The drug loading amount is 408.5 mg of R6G per 1.00 g of Fe_3_O_4_/PAA composited NPs, and the test results are listed in Table 2.

Moreover, the different concentrations (0.15 × 10^−3^, 0.15 × 10^−2^, and 0.15 × 10^−1^ mol/L) of R6G solution are tested to investigate the influence of R6G concentration on drug loading of Fe_3_O_4_/PAA composited NPs. Other experimental parameters are kept the same as the above experiment. As shown in Figure 10C, the amount of drug loading increases along with the improvement of the R6G concentration. However, the concentration could not enhance constantly, as the solubility is limited. Thus, the best concentration of R6G is 0.15 × 10^−1^ mol/L. The detailed results are presented in Table 3. The best drug loading amount is improved to 1011.1 mg per 1.00 g of Fe_3_O_4_/PAA composited NPs.

Finally, the environmental pH values are investigated to understand the influence of drug loading environment on the drug loading process. Four different pH conditions (acetic acid solution (pH = 3.0), sodium hydroxide solution (pH = 10.0), aqueous solution (pH = 7.0) and PBS (pH = 7.4)) are carried out (Figure 10D). The best drug loading amount is obtained in PBS with pH = 7.4. Both low pH and high pH environment do not favor improving the drug loading of these NPs. The comparison results under different solvents are presented in Table 4. 

### 3.9. In Vitro Release Properties of Rhodamine (R6G)

**Figure 11:**
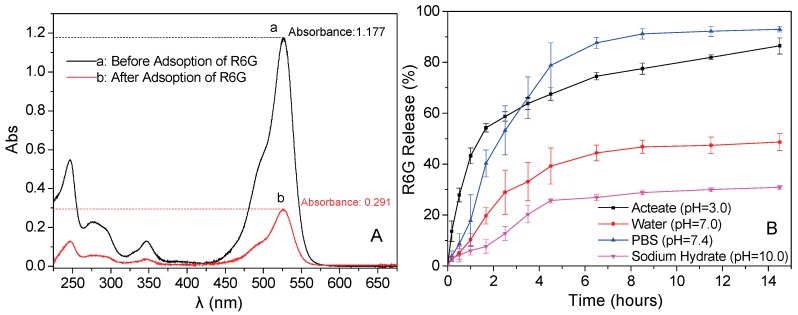
(**A**) UV-Vis spectra of the supernatant before and after absorbing with Fe_3_O_4_/PAA at optimum condition (λ_max_ = 526 nm); and (**B**) cumulative R6G release (%) from Fe_3_O_4_/PAA composite NPs at different pH.

Under optimum conditions, 0.100 g Fe_3_O_4_/PAA NPs are added into 40 mL phosphate buffer solution of R6G (0.15 × 10^−1^ mol/L) for adsorption at 30 °C for 30 min, and then the absorbances of supernatant is measured by diluting them to 10^−4^ magnitude. As shown in Figure 11A, the absorbances of curves (a) and (b) are 1.177 and 0.291, respectively. After calculation, 10.0 mg of drug loaded Fe_3_O_4_/PAA NPs contain 6.851 mg of R6G. Then, 0.010 g of drug loaded Fe_3_O_4_/PAA composited NPs are dispersed into 100 mL of solution with varying pH values at temperature of 37 °C. The absorbances of supernatant are measured by UV-Vis spectra in a certain time interval. Drug release rate of these drug loaded Fe_3_O_4_/PAA composited NPs under different pH condition are calculated and presented in Figure 11B. After releasing for 14 h, the sample in PBS (pH = 7.4) show the best drug release rate, about 93.0% of R6G could be released into the PBS. In aqueous solution (pH = 7.0), about 48.7% of R6G are released in 14 h. It is probably because R6G is a positively charged aqueous solution while carboxylic acid groups on the PAA molecular chains are dissociated to negative carboxylate anions, causing strong molecular electrostatic interactions between R6G and PAA. Therefore, loaded R6G could form ionic bonds with the carriers by the robust electrostatic attraction. Moreover, about 86.5% of the loaded R6G are released within 14 h at pH = 3.0; because more protonated carboxyl groups exist, the R6G could be released from the carrier with the enhancement of the solubility of R6G under acidic conditions. However, in alkaline condition (pH = 10.0), only about 30% of R6G could be released. These results undoubtedly indicate that this drug delivery system composed of Fe_3_O_4_/PAA composited NPs shows a strong pH dependent releasing behavior of R6G. Therefore, as-designed Fe_3_O_4_/PAA composited NPs could potentially be used as drug delivery vehicles for cancer treatment. However, how can we understand such pH dependence and influence related to solvents in DDS? Many chemical and physical questions exist that need to be explored and answered.

### 3.10. Release Mechanism of Rhodamine 6G (R6G)

As the drug releasing results show, the release rate of R6G is different under the various pH environments. The main reason of the phenomenon is proposed as the different solubility between PAA and solvent (or PAA and R6G). For the PAA and solvent, the PAA could be dissolved in the solvent when the solubility parameter of these two substances is the same. The water-solubility of PAA could be calculated using Equation (1), as proposed by P. A. Small [21].

(1) $$\delta =\frac{\sum F_{i}}{V}=\frac{\rho \sum F_{i}}{M}$$

Here, *δ* is the Hansen solubility parameter, *V* is the volume of polymer, and *ρ* and *M* are the density and molecular weight of the polymer repeating unit, respectively. *F_i_* is the molar attraction constant of a specific group *i* according to Equation (1).

In neutral condition and acid condition, in term of PAA, *M* = 72.06, *ρ* = 1.20 g/mL, and *ΣF_i_* = 1399.83, the solubility parameter of PAA can be calculated by above equation, i.e., *δ* = 23.31 (J/cm^2^)^1/2^. The solubility parameter of water is 23.40 (J/cm^2^)^1/2^. It indicates that PAA exhibits good solubility in water because the two solubility parameters are almost identical. Moreover, in term of the model drug R6G, the solubility parameter is 21.77 (J/cm^2^)^1/2^. This solubility parameter is also similar to that of water. Therefore, both R6G and PAA exhibit good solubility in water under neutral condition and acid condition. Meanwhile, R6G can be dissolved and dispersed in the PAA layer, and the swelling effect of PAA on water could promote both the loading and the releasing of R6G [22,23].

By comparison, in alkaline condition, PAA exists as a form of sodium polyacrylate, and *M* = 94.04, *ρ* = 1.32 g/mL, *ΣF_i_* = 1116.23, and *δ* = 15.67 (J/cm^2^)^1/2^. It indicates that PAA shows a low solubility in water. This phenomenon leads to the PAA not being able to be stretched completely in water. Moreover, the solubility of R6G in PAA polymer become worse, and, consequently, the release rate of R6G is lowered evidently. Therefore, in experiment or practical application, R6G is selected as model drug for testing the drug released of Fe_3_O_4_/PAA composite NPs, and services as a significant reference value in drug delivery application. Importantly, solubility parameter is ideal internal reference in DDS.

In Figure 11, we propose a diffusion-driven release dynamics mathematically for the Fe_3_O_4_/PAA composited NPs. The diffusion process of R6G follows the Fick’s second law as Equation (2):

(2) $$\frac{\partial C_{i}}{\partial t}=\frac{D_{ip}\partial^{2}C_{i}}{\partial Z^{2}}$$

Here, *C_i_* is the drug concentration, *t* is time, *D_ip_* is the diffusion coefficient, and *Z* is the position. During the diffusion process, the R6G inside the composite NPs is diffused to a position of its surface, and it can be considered as a kind of membrane diffusion. If *M_t_*/*M_∞_* < 0.6, the equation can be given as Equation (3):

(3) $$\frac{M_{i}}{M_{\infty }}=4{\left(\frac{D_{ip}t}{\pi \delta }\right)}^{1/2}$$

Here, *M_i_* and *M_∞_* denote the absolute cumulative amount of drug released at time *t* and infinity, respectively. *δ* is the thickness of the polymer film. Equation (3) suggests that the relationship of release ratio (*M_i_*/*M_∞_*) and time is not linear. The drug releasing profile derived from the above equation is similar to the parabola in the first quadrant in Figure 11, which is consistent with the results of the experiments. This drug release process accords with the first order kinetics. Thus, the drug releasing process could be controlled to achieve a particular application in biotherapy by changing the morphology of composited NPs, the thickness of polymer layer and the concentration of drug loaded on the composited NPs.

## 4. Conclusions

In summary, PAA-coated hollow Fe_3_O_4_ NPs are successfully synthesized to investigate the function of solubility parameter to drug delivery property. As a result, the coating amount of PAA onto the surface of Fe_3_O_4_ (measured by TGA) is about 40% (*w*/*w*). The best drug release rate was achieved as 93.0% in pH = 7.4 PBS solution after 14 h. The release efficiency is 86.5% in acidic condition. Surficially, it seems that the salt effect and acid effect play significant roles in these two solvents, but it could not explain why a lower release rate (30.0%) is obtained in aqueous solution. Essentially, different forms (polyacrylic acid and polyacrylate) of PAA under variable pH solvents could induce the different solubility parameter of nanocarriers, thus it tunes the coating polymer’s surface solubility, which in turn controls the drug delivery performance of DDS. Moreover, the solubility parameter can also influence the swelling property of PAA, and binding force between PAA and R6G as well. This PAA-coated hollow magnetite DDS provides an example for selecting the solubility parameter as a determining factor in regulation of drug delivery properties, and offers an alternative for controllable DDS which is compatible with both hydrophilic and hydrophobic drug.

## Figures and Tables

**Table 1:** The R6G loading capacity of Fe_3_O_4_/PAA magnetic particles at different temperature.

Temperature (°C)	20	25	30	35	40	50
Absorbance	0.926	0.955	0.860	1.046	1.064	1.159
Drug concentration (×10^−2^ mol/L)	0.115	0.119	0.106	0.130	0.132	0.145
The remaining amount of R6G in 20 mL solution (mg)	11.017	11.355	10.189	12.472	12.693	13.860
The amount of the adsorbed drug by 0.010 g particles (mg)	3.257	2.919	4.085	1.802	1.581	0.414
The amount of the adsorbed drug by 1.0 g particles (mg)	325.7	291.9	408.5	180.2	158.1	41.4

**Table 2:** The Rhodamine 6G (R6G) loading capacity of Fe_3_O_4_/PAA magnetic particles at different adsorptive time.

Adsorption Time (min)	10	20	30	40	50	60
Absorbance	1.109	1.083	0.860	0.964	1.018	1.006
Drug concentration (×10^−2^ mol/L)	0.138	0.135	0.106	0.120	0.127	0.125
The remaining amount of R6G in 20 mL solution (mg)	13.246	12.927	10.189	11.466	12.129	11.981
The amount of the adsorbed drug by 0.010 g particles (mg)	1.028	1.347	4.085	2.808	2.145	2.293
The amount of the adsorbed drug by 1.0 g particles (mg)	102.8	134.7	408.5	280.8	214.5	229.3

**Table 3:** The Rhodamine 6G (R6G) loading capacity of Fe_3_O_4_/PAA magnetic particles at different adsorptive concentration.

Adsorption Concentration (mol/L)	10^−3^	10^−2^	10^−1^
Absorbance	1.004	0.860	0.377
Drug concentration	0.125 × 10^−3^	0.106 × 10^−2^	0.045 × 10^−1^
The remaining amount of R6G in 20 mL solution (mg)	1.198	10.189	42.589
The amount of added magnetic particles (g)	0.001	0.01	0.1
The amount of the adsorbed drug (mg)	0.239	4.085	101.114
The amount of the adsorbed drug by 1.0 g particles (mg)	239.0	408.5	1011.1

**Table 4:** The R6G loading capacity of Fe_3_O_4_/PAA magnetic particles at different pH.

pH	Acetic Acid Solution pH = 3.0	Aqueous Solution pH = 7.0	PBS pH = 7.4	NaOH pH = 10.0
Absorbance	1.054	0.569	0.377	0.866
Drug concentration (×10^−1^ mol/L)	0.131	0.069	0.045	0.107
The remaining amount of R6G in 20 mL solution (mg)	125.501	66.103	42.589	102.508
The amount of the adsorbed drug by 0.010 g particles (mg)	18.202	77.600	101.114	41.195
The amount of the adsorbed drug by 1.0 g particles (mg)	182.0	776.0	1011.1	412.0
